# Effects of fructooligosaccharides on quality attributes of freeze-dried cheese: Insights into water status, microstructure, texture and rheology

**DOI:** 10.1016/j.fochx.2026.104327

**Published:** 2026-08-18

**Authors:** Yang Lyu, Shengnan Li, Lei Gao, Zijian Zhao, Hao Qian, You Kang, Fei Chen, Xindong Jia, Shengyu Li, Yujuan Zhao

**Affiliations:** a Institute of Agro-food Technology, Jilin Academy of Agricultural Sciences (Northeast Agricultural Research Center of China), Changchun 130033, China; b National R&D Center for Milk Processing, Changchun 130033, China; c Xinjiang Shihezi Huayuan Dairy Industry Co., Ltd., Shihezi 832021, China; d Xinjiang Production and Construction Corps Specialty Dairy Processing Technology Innovation Center, Shihezi 832021, China

**Keywords:** Fructooligosaccharides, Freeze-dried cheese, Microstructure, Water status, Quality improvement

## Abstract

Freeze-dried cheese is high-value-added, yet its porous structure and hygroscopicity limit quality and storage stability. This study evaluated the effects of fructooligosaccharides (FOS) on freeze-dried cheese properties. FOS reduced water activity (from 0.13 to 0.08 at 10%FOS) and hygroscopicity, enhancing storage stability. Low-field nuclear magnetic resonance (LF-NMR) and Fourier-transform infrared spectroscopy (FTIR) confirmed enhanced hydrogen bonding after FOS addition, with free water converted to bound water. Scanning electron microscopy (SEM) and micro-computed tomography (μCT) confirmed this redistribution optimized pore morphology and reduced large pores (>150 μm), yielding a dense homogeneous network. These structural improvements increased hardness but reduced brittleness, with 10%FOS delivering the best texture balance. Rheological tests further showed that network strength rose with FOS dosage; tan δ stayed below 0.3 over 25–80 °C. Collectively, FOS supplementation improved the overall quality of freeze-dried cheese, with 10%FOS identified as the optimal level offering superior comprehensive performance and great application potential.

## Highlights

• FOS addition enhances the storage stability of freeze-dried cheese by reducing water activity and hygroscopicity.
• FOS optimizes water distribution and porous structure, enhancing texture, thermal stability, and whiteness.
• FOS enhances the overall quality of freeze-dried cheese through regulating composition and water distribution.

## 1 Introduction

Freeze-drying, which leverages low-temperature and high-vacuum processing (Bhatta et al., 2020), maximally retains the nutritional and sensory attributes of foods (Yang et al., 2023). It imparts a crispy texture and low water activity, thereby extending shelf life (Liu et al., 2022; Zhang et al., 2020). It is widely recognized as an outstanding dehydration technique (Dziki, 2020) that facilitates the development of personalized freeze-dried foods and high-value-added products (Liu et al., 2022). Freeze-drying has been successfully applied to a wide range of food categories, including fruits (Yang et al., 2023), vegetables (Rishabh et al., 2023), dairy products (Silva et al., 2021), and ready-to-eat snacks such as yogurt bites and fruit crisps (Gumul & Kruczek, 2025). However, freeze-dried products face critical challenges. First, their porous structure impairs mechanical strength, leading to brittleness and breakage (Feng et al., 2024). Second, moisture removal often induces physical structural shrinkage or deformation in foods, thereby compromising structural integrity and product quality (Agudelo-Laverde et al., 2014). Finally, the highly porous and amorphous nature of the products renders them extremely sensitive to environmental humidity; upon moisture absorption, they are highly susceptible to structural collapse and quality deterioration (Li et al., 2025). To address these drawbacks, exogenous additives are commonly employed. Recent studies show that hydrocolloids confer a denser porous network in freeze-dried strawberry blocks, enhancing hardness and crispness (Hu et al., 2024). Meanwhile, the incorporation of biopolymers as fillers effectively reduces the hygroscopicity of freeze-dried restructured jujubes (FDRJ) while improving their loose, porous internal structure (Li et al., 2025). Additionally, adjusting the ratio of different polysaccharides can modulate the pore structure, mechanical strength, and hydration behavior of the freeze-dried matrix, thereby influencing the textural properties and microstructure of the final freeze-dried products (Feng et al., 2023).

Cheese is renowned for its rich nutritional value and diverse sensory attributes, holding a prominent position in global dietary cultures (Akbar et al., 2025). Consumer demand for convenience foods has driven the food industry to explore diverse processing technologies. With advancements in food processing techniques, the variety and forms of cheese snacks have become increasingly diversified. For instance, crispy pure cheese puffs have been produced via microwave vacuum drying technology (Chudy et al., 2019), and mozzarella cheese snacks have been prepared by microwave vacuum drying (Simão et al., 2025). However, high-moisture, hard, and ripened cheeses each have distinct limitations for freeze-drying; a fresh rennet-coagulated cheese was therefore used as a simplified model. As a high-value-added dairy product, freeze-dried cheese combines the nutritional benefits of cheese with the convenience of freeze-dried foods, exhibiting substantial market potential. However, previous studies have primarily focused on process optimization and pre/post-drying quality comparisons (Köprüalan Aydın et al., 2025; Paul et al., 2020), lacking systematic investigation into how matrix component changes affect final quality, as well as a lack of proactive control strategies based on such understanding. Therefore, the incorporation of exogenous additives to actively regulate microstructure formation and phase distribution during cheese freeze-drying represents a promising approach to improve the texture and overall quality of freeze-dried cheese.

Existing research indicates that FOS, through its strong hydrophilicity and hydrogen-bonding interactions, can regulate water distribution (Su et al., 2020; Guo, Huang, Chen, Lei & Lu, 2025), enhance protein network interactions (Wang et al., 2024), and improve phase transition behavior and ice crystal growth patterns during freeze-drying (Feng et al., 2024; Guerreiro et al., 2020; Sun et al., 2022). These functions collectively refine microporous morphology and promote the assembly of homogeneous, compact porous matrices. FOS was selected for its prebiotic benefits, low molecular weight, good solubility, strong hydrophilicity, and commercial availability. However, mechanistic insight remains lacking into how FOS remodels the microstructure and textural attributes of freeze-dried cheese. Cryoprotection research on polysaccharides largely relies on carbohydrate-rich plant gels, fruits, and dough systems. In contrast, freeze-dried cheese is based on a fat-containing coagulated casein network, so results derived from plant-based matrices cannot be reliably extrapolated to dairy systems. Furthermore, prior investigations of freeze-dried cheese have largely focused on drying process optimisation, with little attention paid to tuning matrix properties using FOS. In this study, different doses of FOS were added to cheese followed by freeze-drying. We systematically investigated the effects of FOS incorporation on key quality attributes of freeze-dried cheese, including moisture distribution, microstructure, pore size distribution, texture and rheology. The interaction mechanism between FOS and the cheese matrix was elucidated, with the aim of providing theoretical basis and technical support for the development of high-quality freeze-dried cheese products.

## 2 Materials and methods

### 2.1 Cheese manufacture and experimental design

The cheeses were made according to the method of Deshwal et al. (2020) with minor modifications. Four batches were designed: a control (CON) and three FOS (≥ 90% *w*/w, average DP > 10, Macklin Biochemical, Shanghai, China) supplementation levels—5%, 10% and 15% (*w*/w, milk basis) denoted as 5%FOS, 10%FOS and 15%FOS, respectively. For each batch, FOS was dissolved directly in 5 kg of milk (standardized to 3.5% *w*/w fat and 3.1% *w*/w protein) at 65 °C and pasteurized for 30 min then cooled to 37 °C and then inoculated with 0.25 g of commercial calf rennet (activity 200 IMCU/g, Doit Biotechnology, Italy). Coagulation proceeded for 60 min, after which the curd was cut into 1 cm^3^ cubes and heated to 40 °C hot broth for 30 min with gentle stirring, followed by the expulsion of the whey. Then, the cheese curd was transferred to the molds and pressed under 0.3 MPa for 2 h, vacuum-packed in polyethylene pouches and stored at −40 °C until completely frozen for subsequent freeze-drying. A fresh, non-fermented rennet-coagulated cheese was prepared as an experimental model system. This simplified system was specifically chosen to evaluate the direct effects of FOS addition on freeze-drying behavior, excluding the confounding influences of microbial fermentation and ripening-associated proteolysis. We acknowledge that this single cheese type may limit the generalizability of the findings, and further studies with ripened cheese varieties are warranted. For each formulation, three independent cheese-making batches were performed. All processing parameters, including milk source, pasteurization conditions, rennet concentration, coagulation time and pressing procedure, were strictly standardized across batches to minimize batch-to-batch variability.

### 2.2 Freeze-drying of cheese blocks

Cheese samples were cut into 1 cm^3^ cubes and freeze-dried using a vacuum freeze dryer (FDU-2110, EYELA, Japan). The cubes were frozen at −40 °C for 4 h, then dried with a cold trap at −80 °C and a pressure of 0.998 mbar for 72 h.

### 2.3 Content of FOS

The content of FOS was determined using high-performance anion-exchange chromatography coupled with pulsed amperometric detection (HPAEC-PAD). Approximately 50 mg of freeze-dried cheese sample was accurately weighed, added to 0.7 mL of 80% ethanol solution, and shaken in a 50 °C water bath for 2 h. Subsequently, 0.7 mL of ultrapure water was added for dilution. After centrifugation, the supernatant was filtered and reserved for analysis. The Thermo ICS 5000+ ion chromatography system (ICS5000+, Thermo Fisher Scientific, USA) was employed, equipped with a Dionex™ CarboPac™ PA20 (150 × 3.0 mm, 10 μm). Mobile phase A was ultrapure water, B was 0.1 M NaOH, and C was 0.1 M NaOH +0.2 M NaAc. The flow rate was 0.4 mL/min, column temperature was 30 °C, and injection volume was 5 μL. Gradient elution was employed to separate target components. Quantification was carried out by external standard calibration using authentic standards, with calibration curve concentrations ranging from 1 to 10 μg/mL. Data acquisition and processing were performed with Chromeleon software, and the absolute content of each FOS component in the sample was calculated based on peak area. (Ispiryan et al., 2019).

### 2.4 Cheese composition and yield

Moisture content was determined by oven-drying 2 g of ground cheese at 105 °C for 24 h and expressed as weight loss percentage. Protein content was analyzed using the Kjeldahl method (0.5 g sample, nitrogen-to-protein conversion factor 6.38). Fat content was measured by Soxhlet extraction (AOAC International, 2023) with petroleum ether for 6 h (2 g sample). Ash content was determined by incineration at 550 °C (2 g sample). Cheese yield was calculated as the percentage of freeze-dried solids obtained per unit of raw milk weight (g/100 g milk).

### 2.5 Determination of water status

#### 2.5.1 Water activity

Water activity (aw) of freeze-dried cheese was measured at 25 °C with a dew-point hygrometer (HD-5, Yima, China). Each set of samples was measured three times.

#### 2.5.2 Hygroscopicity

Approximately 5 g of freeze-dried cubes were placed in a desiccator maintained at 25 °C and 75% relative humidity using a saturated NaCl solution. The samples were kept in the desiccator until the mass change was <0.01 g over 48 h (Yang et al., 2023). Hygroscopicity was expressed as the percentage mass gain:

Hygroscopicity (%) = [(mass after exposure - initial mass) / initial mass] × 100.

#### 2.5.3 Rehydration ratio

The freeze-dried cheese was placed in distilled water at around 30 °C to absorb water thoroughly for 30 min, after which the surface moisture of the sample was blotted dry with filter paper (Feng, Bi, Yi, Li, Li, & Ma, 2022). Rehydration ratio was calculated as:

Rehydration ratio (%) = (mass after rehydration / mass before rehydration) × 100.

#### 2.5.4 LF-NMR analysis

Water distribution and mobility in freeze-dried cheese were assessed on a 20 MHz low-field NMR spectrometer (MesoMR23-060H, Niumag, China; 0.5 T) at 25 °C. Freeze-dried cheese was placed into 25 mm-diameter NMR tubes for measurement. A CPMG sequence was used: TE 0.3 ms, TR 1600 ms, 50000 echoes, 8 scans. Each set of samples was measured three times. The signal obtained from the CPMG sequence was inverted using simultaneous iterative reconstruction technique (SIRT) algorithm to obtain a detailed inversion spectra of T_2_.

### 2.6 Microstructure analysis

#### 2.6.1 X-ray diffraction (XRD)

The crystal structures of freeze-dried cheese were determined using an X-ray instrument (D8 Advance, Bruker, Germany). The sample was ground into a uniform powder and evenly spread it on the sample stage. At ambient temperature, the samples were scanned in the 2θ range of 5–90° with scanning speed of 2°/min.

#### 2.6.2 Fourier-transform infrared spectroscopy (FTIR)

Functional groups were identified using an FT-IR spectrometer (Nicolet iS20, Thermo Fisher Scientific, USA). Freeze-dried samples were cryo-ground under liquid nitrogen to <50 μm particles, mixed with KBr (1:100, *w*/w) and compressed into translucent pellets. Spectra were acquired from 400 to 4000 cm^−1^ at 4 cm^−1^ resolution averaged over 32 scans.

#### 2.6.3 Scanning electron microscopy (SEM)

Following the protocol of Liu et al. (2021) with minor adjustments, the microstructure of freeze-dried cheese was visualised by scanning electron microscopy (FE-SEM, ZEISS Sigma 300, ZEISS, Germany). Cubes (1 × 1 × 1 cm^3^) were fixed to aluminium stubs with double-sided carbon tape and sputter-coated with a 10 nm Au—Pd (80:20) layer (IB-3, Eiko, Japan) to ensure electrical conductivity. Samples were examined at an accelerating voltage of 15 kV and a working distance of 8 mm. Micrographs were recorded at 1800× magnifications, with three randomly selected fields per specimen to ensure reproducibility.

#### 2.6.4 Micro-computed tomography (μCT)

X-ray μCT was performed on freeze-dried cubes (1 cm^3^) using a nanoVoxel 1000 system (Sanying Precision Instrument, China) operated at 50 kV and 200 μA. Samples were secured to the holder (source-to-sample distance = 78.1 mm) with polyimide tape to minimize motion artefacts. A total of 1440 projections were obtained over a 360° rotation at 9.5 μm isotropic voxel size. Raw projections were reconstructed into a 3-D volume and analyzed in VG Studio MAX to quantify porosity, pore-size distribution and other morphometric parameters. Each sample was analyzed in triplicate.

### 2.7 Texture analysis

Hardness, fracturability and chewiness were determined using a TA.XTplus texture analyzer (Stable Micro Systems, UK) equipped with a 35 mm diameter cylindrical probe (P/35). Both fresh and freeze-dried samples were cut into 1 cm^3^ cubes and equilibrated to 25 °C. Tests were run in compression mode to 30% strain at 1.00 mm/s, with pre- and post-test speeds of 2.00 mm/s and a trigger force of 10.0 g. Each sample was analyzed in triplicate.

### 2.8 Rheological analysis

Rheological properties of freeze-dried cheese were measured with a controlled-stress rheometer (DHR-2, TA Instruments, USA) using 25 mm parallel-plate geometry (gap = 2 mm). Prior to testing, strain scanning tests were conducted at a constant frequency of 1 Hz to determine the linear viscoelastic region (LVR). For frequency scanning testing, the frequency range was set to 0.1–100 rad/s, the strain amplitude parameter was selected as 0.02%, and the storage modulus (G'), loss modulus (G"), and tan δ (the ratio of G" to G') were measured. The sample underwent controlled temperature scanning from 25 °C to 80 °C at a constant heating rate of 2 °C/min (Rogers et al., 2010), maintaining a predetermined LVR strain range (constant frequency of 1 Hz and constant amplitude strain of 0.01%), to monitor how G', G", and tan δ changed with increasing temperature. Each sample was analyzed in triplicate.

### 2.9 Color determination

The color of freeze-dried cheese was measured using a colorimeter (NS808, Shenzhen Threenh Technology Co Ltd., China) based on the CIE Lab* color space. Three replicates were measured per sample, and the average L*, a*, and b* values were recorded.

### 2.10 Statistical analysis

Each experiment was performed in three independent batches. Results are presented as mean ± SD. Normality was checked using the Shapiro–Wilk test, and homogeneity of variances was verified using Levene's test prior to ANOVA. Statistical analysis was conducted using SPSS software (version 26.0). Tukey's honest significant difference test was used for post-hoc multiple comparisons to identify specific group differences. Data processing and graph rendering were performed using Origin software 2021. Pearson's correlation coefficient was performed using the packages “Correlation Plot” packages in Origin software 2021. A *p*-value of less than 0.05 was set as the threshold for statistical significance, ensuring robust interpretation of the impact of varying FOS concentrations on cheese characteristics.

## 3 Results and discussion

### 3.1 Effect of FOS addition on the composition and yield of freeze-dried cheese

The gross composition and yield of freeze-dried cheeses with 0–15% FOS are summarised in Table 1. FOS content in freeze-dried cheese correlated positively with its addition level, indicating effective retention during processing. This can be attributed to the neutral and low-molecular-weight nature of FOS, which only forms weak reversible hydrogen bonds with casein and is mostly washed away with whey during curd drainage. A high initial FOS supplementation level in milk was therefore adopted to guarantee sufficient FOS retention in curds for regulating the quality of freeze-dried cheese. Typically, the final moisture content of freeze-dried materials ranges from 0.5% to 3%, as higher values can compromise long-term storage stability (Merivaara et al., 2021). The moisture contents of the four groups of freeze-dried cheese fell within this reasonable range. Compared with the control (30.60 g 100 g^−1^), the protein content initially increased in the 5%FOS group (33.10 g 100 g^−1^), but then decreased in the 10%FOS and 15%FOS groups (28.70 and 25.90 g 100 g^−1^, respectively). This initial increase may be attributed to concentration rather than true enrichment, because the added FOS displaces fat but not protein at this low dosage. At higher inclusion rates, abundant hydroxyl groups on FOS compete for hydrogen bonding with casein micelles, altering protein aggregation and reducing the measurable protein fraction in the dried matrix (Wang et al., 2024). In addition, fat and ash contents were inversely related to FOS content, consistent with previous findings (Chailangka et al., 2023). The yield of freeze-dried cheese significantly increased with FOS addition (*p* < 0.05). Cheese yield is related to coagulation (Vacca et al., 2020), and the addition of FOS may have promoted the coagulation process through a synergistic effect. Silva et al. (2021) noted that when the charge characteristics of polysaccharides are appropriate, they could play a positive role in the bond rearrangement of casein aggregates rather than causing interference, leading to more casein involvement in synergy and consequently increasing yield.

**Table 1:** Chemical composition and yield of freeze-dried cheese supplemented with varying levels of FOS.

Sample	CON	5%FOS	10%FOS	15%FOS
FOS (g/100 g)	n.d.	0.86 ± 0.02^c^	1.14 ± 0.01^b^	1.29 ± 0.02^a^
Moisture (g/100 g)	2.20 ± 0.20^ab^	2.40 ± 0.25^ab^	2.50 ± 0.17^a^	2.10 ± 0.35^b^
Protein (g/100 g)	30.60 ± 0.44^b^	33.10 ± 0.26^a^	28.70 ± 0.36^c^	25.90 ± 0.20^d^
Fat (g/100 g)	56.80 ± 0.70^a^	54.10 ± 0.10^b^	51.10 ± 0.17^c^	48.10 ± 0.36^d^
Ash (g/100 g)	3.20 ± 0.20^a^	2.70 ± 0.10^b^	2.40 ± 0.20^b^	1.90 ± 0.10^c^
Yield (%)	6.47 ± 0.16^d^	7.22 ± 0.07^c^	7.60 ± 0.22^b^	8.10 ± 0.19^a^

1 n.d. = not determined (FOS was not added to this formulation).

2 Values are expressed as mean ± SD, *n* = 3.

3 a-d Values followed by different letters in the same row are significantly different (*p* < 0.05); values with the same letter are not significantly different.

### 3.2 Water status of the freeze-dried cheese

#### 3.2.1 FOS addition lowers water activity, hygroscopicity and rehydration ratio of freeze-dried cheese

Table 2 demonstrates that incremental FOS supplementation progressively reduced water activity (aw), hygroscopicity and rehydration capacity of freeze-dried cheese. The aw declined from 0.13 (CON) to 0.08 upon incorporation of ≥10%FOS (*p* < 0.05). This may be attributed to the strong hydrophilicity of FOS, which preferentially bind water molecules, thereby reducing water activity(Guo, Huang, Chen, Wu & Lu, 2025). Notably, both 10% and 15%FOS showed identical aw (0.08), which could be due to the saturation of hydrogen-bonding sites, similar to the hydrogen-bonding-mediated saturation phenomena observed in milk protein-polyphenol systems (van de Langerijt et al., 2023). Concomitantly, hygroscopicity decreased from 31.47% to 22.86%, implying that while residual moisture is more tightly retained, the matrix absorbs less atmospheric water. Hygroscopicity is determined by sample composition and microscopic morphology. On the one hand, the hydrogen bond cross-linking between FOS and water molecules restricts the mobility of moisture within the samples by converting free water into a more tightly bound state (Hu et al., 2024). Meanwhile, the incorporation of FOS results in the formation of a denser porous structure, with a corresponding decrease in porosity. This structural modification effectively reduces the migration rate of ambient moisture toward the sample via the capillary effect (Hu et al., 2024). Previous studies have indicated that pore structure also exerts a significant influence on the rehydration behavior of samples (Zhang et al., 2024). Consistent with this, the addition of FOS in the present study notably reduced the sample rehydration ratio, which was 17% lower than that of the control group (*p* < 0.05). Such a reduction can be attributed to the decreased porosity and diminished open structure, which impaired the matrix's capacity to capture water during the rehydration process (Zhang et al., 2024). These findings are further supported by subsequent μCT analysis.

**Table 2:** Water activity, hygroscopicity and rehydration ratio of freeze-dried cheese supplemented with different levels of FOS.

Sample	Water activity	Hygroscopicity (%)	Rehydration ratio (g/g)
CON	0.13 ± 0.00^a^	31.47 ± 0.81^a^	2.00 ± 0.15^a^
5%FOS	0.12 ± 0.00^b^	26.30 ± 0.77^b^	1.82 ± 0.04^b^
10%FOS	0.08 ± 0.00^c^	23.81 ± 0.15^c^	1.73 ± 0.05^b^
15%FOS	0.08 ± 0.00^c^	22.86 ± 0.59^c^	1.66 ± 0.04^b^

1 Values are expressed as mean ± SD, *n* = 3.

2 a-d Values followed by different letters in the same column are significantly different (*p* < 0.05); values with the same letter are not significantly different.

#### 3.2.2 Low-field NMR revealed FOS-induced water redistribution in freeze-dried cheese

**Fig. 1:**
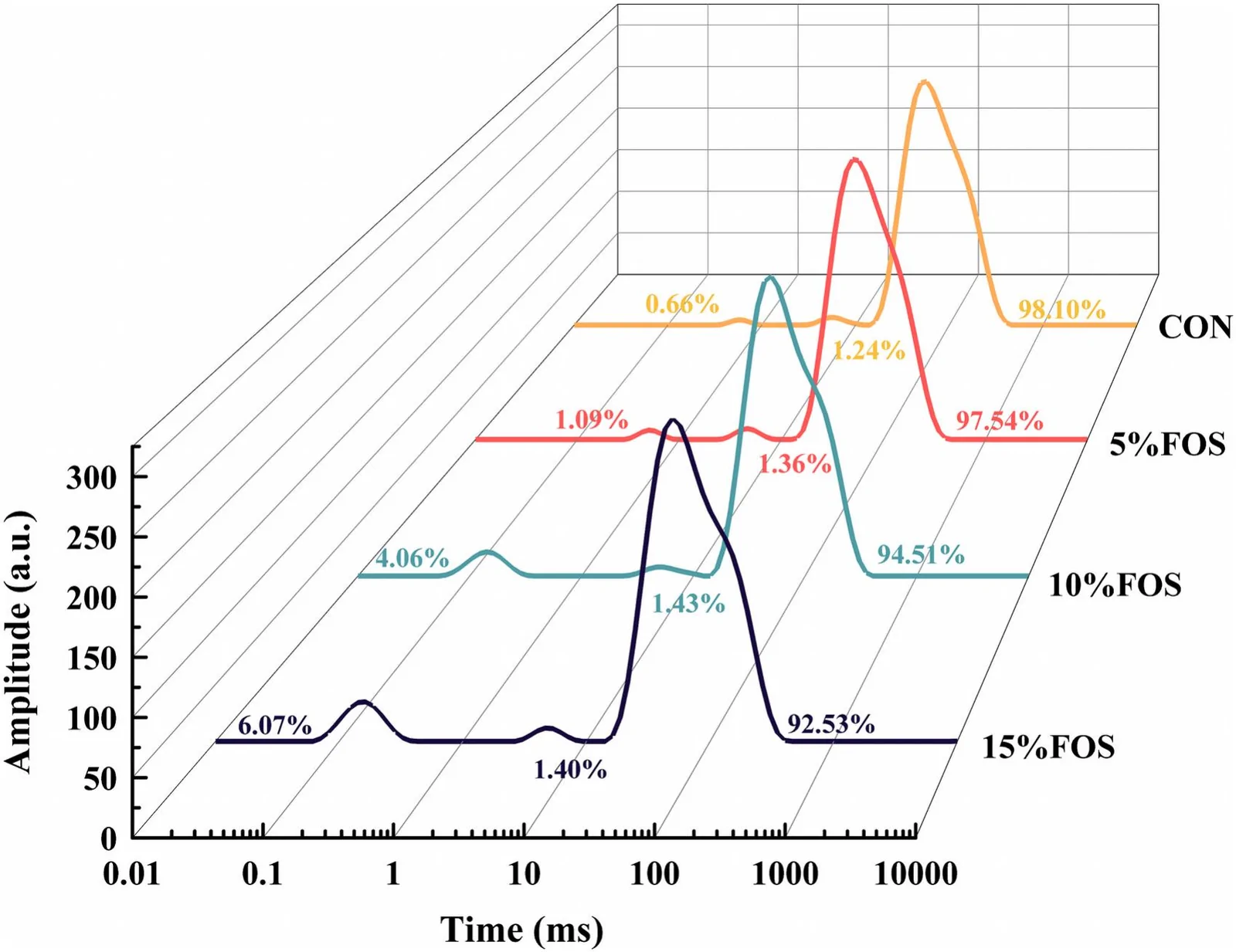
Low-field ^1^H NMR T₂ relaxation spectra of freeze-dried cheese supplemented with different levels of FOS.

Low-field ^1^H NMR distinguishes water populations by their transverse relaxation time (T₂): shorter T₂ indicates stronger water–matrix interactions and lower proton mobility, whereas longer T₂ corresponds to more mobile, easily removed water (Hu et al., 2024). Three T₂ components were isolated (Fig. 1): T₂₁ (0.1–1 ms), T₂₂ (1–10 ms), and T₂₃ (10–100 ms), representing bound water, immobilized water, and free water, respectively (Younas et al., 2021). The addition of FOS resulted in a shortening of T₂₁ and a leftward shift of T₂₃ toward lower relaxation times. These shifts indicate reduced proton mobility, consistent with enhanced water–matrix interactions that result in a more tightly bound water state with decreased molecular freedom, likely arising from hydrogen bonding between the hydroxyl groups of FOS and water molecules (Liu et al., 2025; Wang et al., 2023). Concurrently, the proportional peak area of T₂₁ increased while that of T₂₃ decreased with increasing FOS concentration. This suggests that a portion of the free water population was converted into a more tightly bound state. This redistribution is presumably driven by the strong hydrophilic nature of FOS, which competes for water molecules and restricts their mobility within the cheese matrix (Liu et al., 2025). In other words, FOS interferes with the binding between the cheese matrix and water, driving a phase transition of water within the system, which may further influence the microstructure and texture properties of the freeze-dried cheese.

### 3.3 XRD analysis

**Fig. 2:**
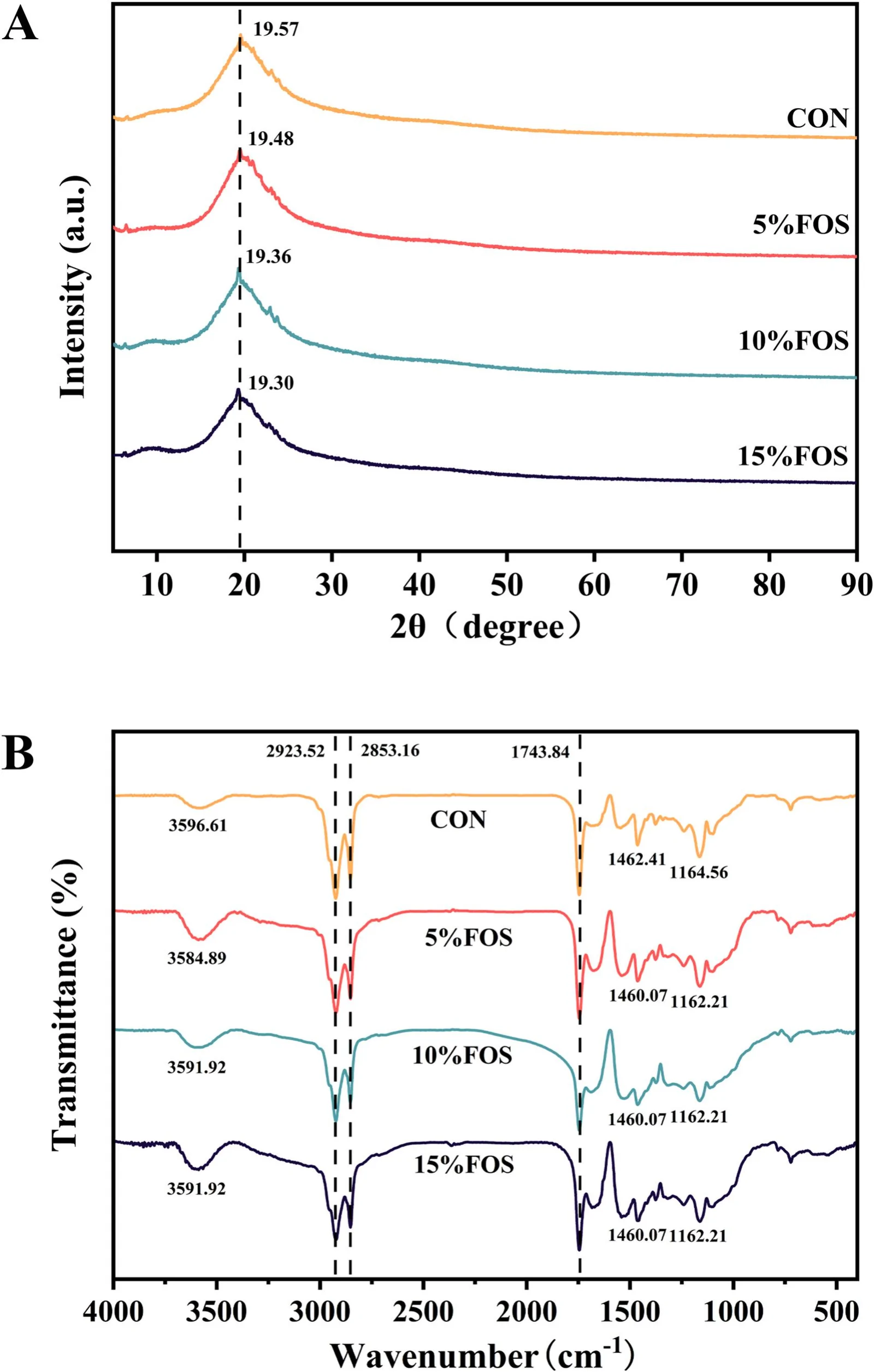
XRD pattern (A) and FTIR spectra (B) of the freeze-dried cheese supplemented with different levels of FOS.

XRD was employed to characterize changes in the crystal structure and intermolecular interactions of freeze-dried cheese samples. The diffraction patterns for different FOS levels are shown in Fig. 2A. All samples exhibited a broad halo centered at 2θ ≈ 19.5°, indicating that the cheese matrix remained predominantly amorphous irrespective of FOS addition. This indicates that FOS did not induce any new crystalline phases. Such behavior is consistent with the known freeze-drying behavior of sugars: the cryoconcentration effect during freezing drives vitrification, which kinetically traps sugar molecules and prevents their recrystallization, so FOS stays amorphous in the final dried matrix. Compared with the control, FOS-supplemented samples showed a slight shift of the diffraction peak toward lower 2θ angles and a decrease in peak intensity. These changes probably arise from hydrogen-bonding interactions between FOS and water molecules (Su et al., 2020). The strong hydrophilic affinity of FOS retains bound water within the cheese matrix (Li et al., 2022); such water molecules may intercalate between crystal planes, effectively increasing the interplanar spacing (Guo, Huang, Chen, Wu & Lu, 2025). Similar observations have been reported for oligosaccharides in freeze-dried systems, where the amorphous phase is preserved, giving broader and weaker diffraction peaks (Deng et al., 2023).

### 3.4 FTIR analysis

FTIR spectroscopy was used to analyze molecular structural changes and interactions in cheese induced by FOS addition during freeze-drying (Fig. 2B). No new characteristic peaks appeared in FOS-added samples compared to the control, indicating no new covalent bond formation (Zhang et al., 2019).

However, all four samples exhibited a stretching vibrational peak of -OH at approximately 3600 cm^−1^, and the addition of FOS to the cheese resulted in a shift of the absorption peak at 3600 cm^−1^ to a lower wavenumber. Specifically, the -OH stretching peak shifted from 3596.61 cm ^−1^ in the CON group to 3591.92 cm ^−1^ in the 15%FOS group. In freeze-dried cheese, this absorption peak originates from the stretching vibrations of hydroxyl groups in casein and FOS (Paul et al., 2020), suggesting that FOS addition enhanced intramolecular and intermolecular hydrogen bonding (Chandrakar et al., 2024). This may be attributed to the abundant hydroxyl groups in FOS, which can serve as potential hydrogen bond donors and acceptors (Lou et al., 2024). Additionally, FOS molecules may occupy spatial sites on the protein surface and interact with charged residues (e.g., carboxyl and amino groups) through hydrogen bonding (Santiago-García et al., 2021). The absorption peak at 1745 cm^−1^ corresponds to the stretching vibration of C=O (carbonyl groups) in casein. As shown in Fig. 2B, FOS addition did not cause a shift in the peak position. The absorption peak at 1462 cm^−1^ represents the bending vibration of methyl groups (CH₃) in fat molecules (Di Donato et al., 2022). FOS addition caused a red shift of the CH₃ peak from 1462.41 cm^−1^ to 1460.07 cm^−1^. Additionally, the absorption peak in the “fingerprint region” of sugars (corresponding to C-O-C stretching vibrations) (Di Donato et al., 2022) shifted to a lower wavenumber (from 1164.56 cm^−1^ to 1162.21 cm^−1^) compared with the CON group, which was associated with FOS addition. These spectral changes are consistent with the presence of hydrogen bonding interactions between FOS and the molecular components of freeze-dried cheese. In conclusion, the FTIR data suggest that different FOS addition ratios modulate intermolecular interactions in freeze-dried cheese, potentially inducing changes in its internal structure. However, it should be noted that FTIR provides indirect evidence of molecular associations, and complementary techniques would be required to definitively characterize specific interaction types.

### 3.5 Effect of FOS on the microstructure of freeze-dried cheese

**Fig. 3:**
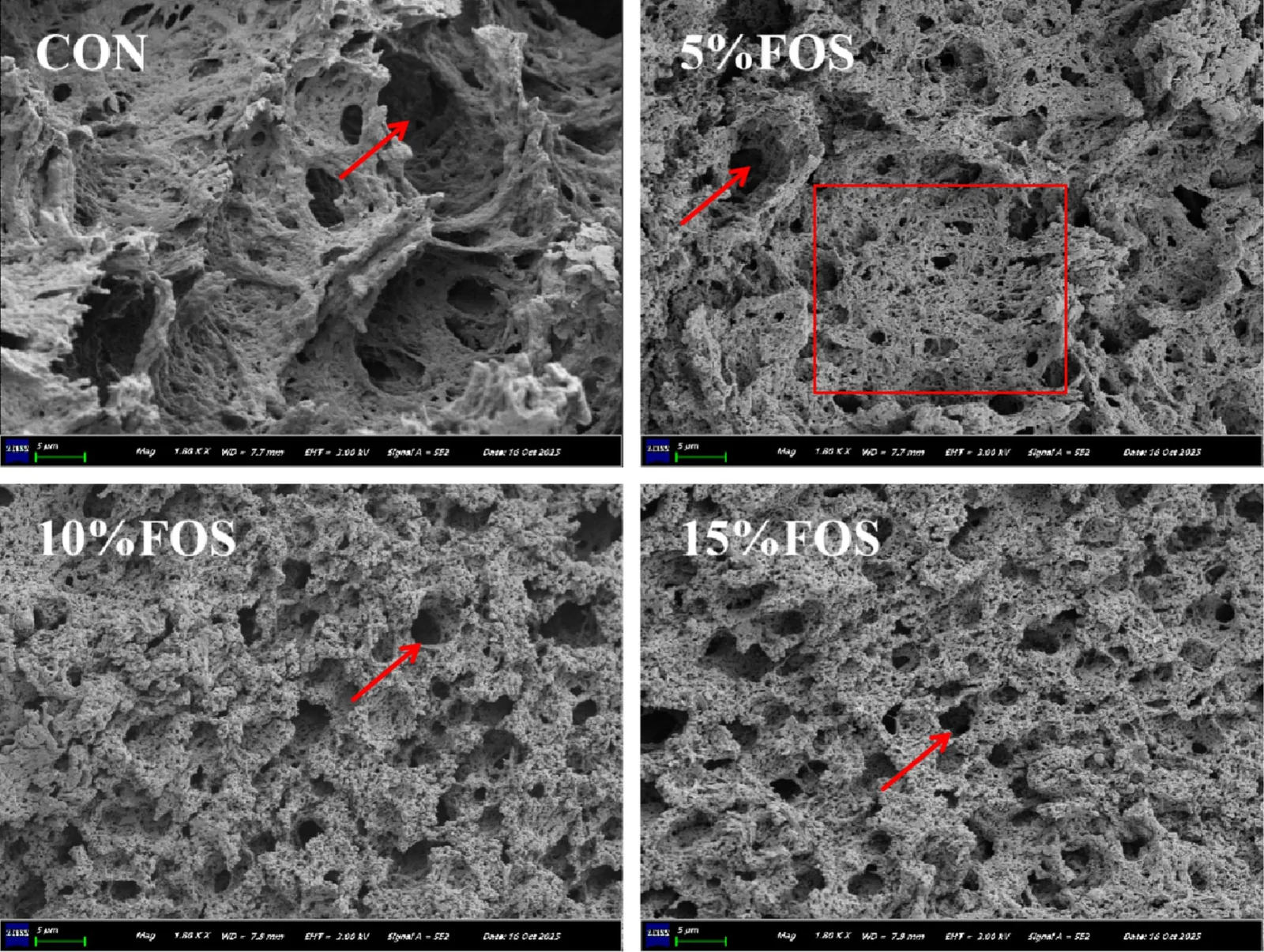
Scanning electron microscopy images (×1800) of the freeze-dried cheese supplemented with different FOS levels.

Fig. 3 shows SEM images of freeze-dried cheese with different levels of FOS addition. As highlighted by the arrows in Fig. 3, the control group (CON) exhibits a coarse and heterogeneous porous architecture characterized by large, irregular pores with thick walls. Upon 5%FOS addition, porosity and pore size are visibly reduced; however, local heterogeneity and a loosely knit framework remain evident. This compacting effect is amplified at 10% and 15%FOS, yielding a more ordered and microporous microstructure with thinner pore walls and a more homogeneous solid network. Previous studies have reported similar pore-refining effects when oligosaccharides or hydrocolloids were incorporated into freeze-dried food matrices (Feng, Bi, Yi, Li, Lyu, et al., 2022). The refined pore structure observed in FOS-supplemented samples is likely related to the dual role of FOS in modulating ice crystallization. On the one hand, the abundant hydroxyl groups of FOS may serve as effective heterogeneous nucleation sites, potentially promoting the formation of numerous small ice crystals during freezing (Guerreiro et al., 2020; Sun et al., 2022). On the other hand, the strong water-binding capacity of FOS could trap free water via hydrogen bonding, likely curtailing molecular migration to the ice-crystal front and limiting crystal growth (Sun et al., 2022). The resulting smaller, more evenly distributed ice crystals would then translate into a denser cheese matrix upon sublimation.

### 3.6 Macroscopic porous structure of the freeze-dried cheese

**Fig. 4:**
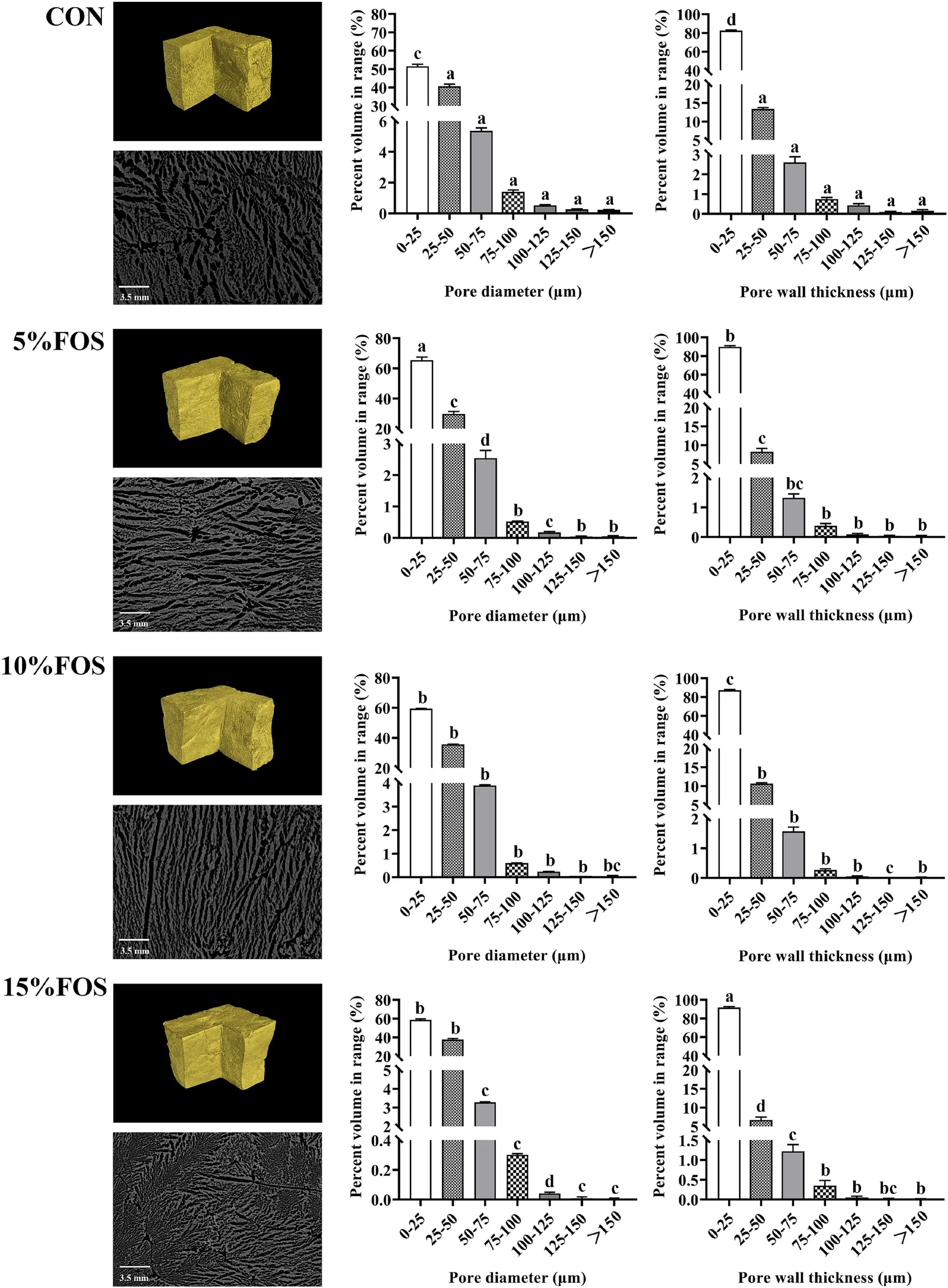
μCT reconstructed 3D sample images, 2D front views, pore diameter distribution, and pore wall thickness distribution of freeze-dried cheese supplemented with different FOS levels. All aperture distributions and pore wall thickness maps are plotted at a uniform y-axis scale for direct visual comparison between groups. Segmented y-axes in certain panels were used to better visualize the extremely low proportions of large pores (>100 μm) in FOS-supplemented groups, which would otherwise be barely discernible under a fully unified scale. Different letters (a–d) above bars within the same interval denote significant differences among the four FOS groups (*p* < 0.05, *n* = 3).

Micro-computed tomography (μCT) imaging (Fig. 4) provides clear visualization of the microstructure of freeze-dried cheese at different FOS levels. Three-dimensional (3D) reconstructions confirmed that all samples retained their structural integrity, with no evidence of collapse or deformation. The 3D reconstructed models further revealed a sponge-like porous structure, in which the bright continuous phase represents the cheese matrix and dark regions correspond to discrete pores (Frisullo et al., 2012; Schoeman et al., 2016). The control showed large, heterogeneously distributed pores; as FOS supplementation increased, the pore network became progressively refined and more homogeneous, with the 10% and 15%FOS groups displaying densely distributed dark regions and markedly smaller pore sizes in the slice images. These results indicate that FOS not only prevents local structural collapse during freeze-drying but also effectively refines the internal pore architecture.

Quantitative pore-structure parameters derived from the two-dimensional (2D) slices are also shown in Fig. 4, revealing distinct differences among groups. FOS supplementation increased the proportion of micropores (0.1 < d < 100 μm) (Gun'ko, Savina & Mikhalovsky, 2013), while the proportion of large pores (d > 150 μm) decreased progressively (CON: 0.23 ± 0.02%; 5%FOS: 0.05 ± 0.02%; 10%FOS: 0.04 ± 0.03%; 15%FOS: 0.01 ± 0.00%), confirming that higher FOS concentrations (10% and 15%) produced a more uniform and denser matrix. The pore-wall thickness distribution followed a similar trend: all FOS-added samples had an increased proportion of thin walls (0–100 μm), accounting for 99.89% of the total wall population, indicating that FOS promotes the formation of thinner pore walls. Earlier studies have also reported that polysaccharide addition can create a more compact and uniform porous network in freeze-dried strawberry matrices (Wang et al., 2025).

### 3.7 Texture analysis

**Fig. 5:**
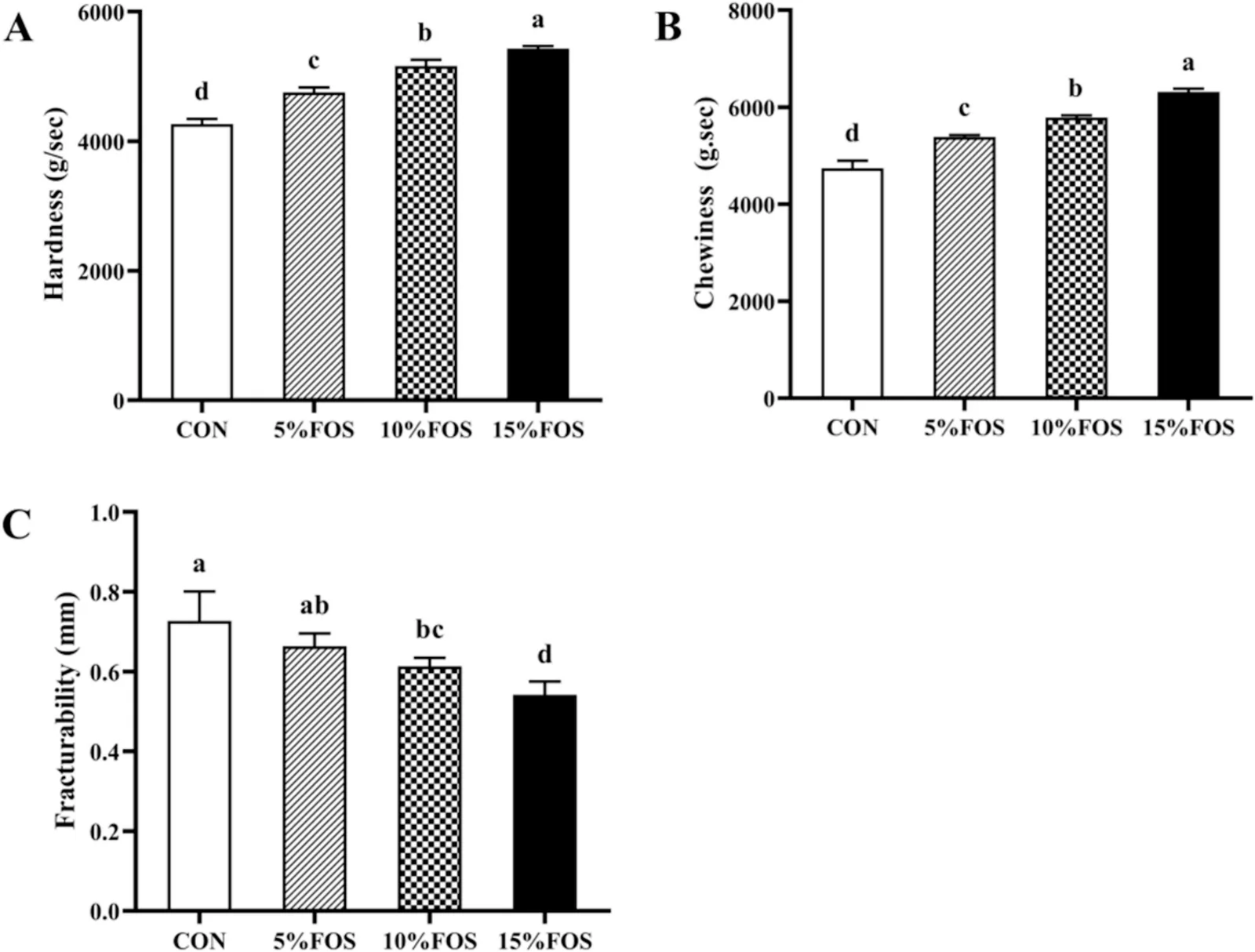
Textural parameters of freeze-dried cheese supplemented with different levels of FOS. (A) Hardness, (B) Chewiness, (C) Fracturability. Different lowercase letters (a–d) above the bars indicate significant differences among groups (*p* < 0.05).

We evaluated the texture characteristics of freeze-dried cheese with different FOS addition levels based on hardness, chewiness and fracturability. As shown in Fig. 5A and 5B, compared with the control group, as the FOS addition level increased from 5% to 15%, the hardness and chewiness of the cheese exhibited trends consistent with the FOS addition level, increasing significantly from 4264.51 ± 79.97 g/s and 4742.70 ± 151.72 g·*sec* to 5428.84 ± 39.89 g/s and 6315.28 ± 64.41 g·*sec*, respectively. Previous studies have demonstrated that incorporating hydrophilic components as functional fillers is an effective strategy for enhancing the strength of biopolymer networks. For example, in gluten systems, the hydrophilic nature of inulin enables it to fill interstitial spaces within the network and reinforce the structure, thereby significantly improving its strength (Yue et al., 2025). Regarding fracturability, among all formulations, the 10%FOS group achieved the most favorable balance, showing the highest hardness while maintaining moderate fracturability, though its fracturability was significantly lower than that of the CON group (*p* < 0.05) (Fig. 5C). This decline is attributable to the denser, more homogeneous microstructure formed upon FOS incorporation (Fig. 3, Fig. 4), which reduces the number of large pores and critical flaw sites that would otherwise act as stress concentrators and facilitate crack propagation (Nimmo, 2004; Zhao et al., 2018).

The refined microporous network increased the solid volume fraction and enhanced the resistance to compressive deformation, leading to greater hardness. At the same time, the smaller pore sizes and thinner pore walls reduced the available pathways for energy dissipation during fracture (Cao et al., 2025), so that once a crack was initiated, it propagated rapidly across the matrix, yielding a lower measured fracturability. Thus, the simultaneous rise in hardness and fall in fracturability reflects a progressive structural densification rather than a contradiction. This mechanical behavior, characterized by strong resistance to deformation yet easy fracture upon reaching a critical stress, is typical of a hard and brittle texture, which is often advantageous for freeze-dried products that must endure handling while remaining easily breakable at the point of consumption (Feng et al., 2024). Nevertheless, exclusively prioritizing one attribute over the other might not be a prudent strategy for optimizing texture quality (Feng et al., 2024). Among all formulations, the 10%FOS group achieved the most favorable balance, showing the higher hardness while preserving moderate fracturability.

### 3.8 Rheological properties analysis

**Fig. 6:**
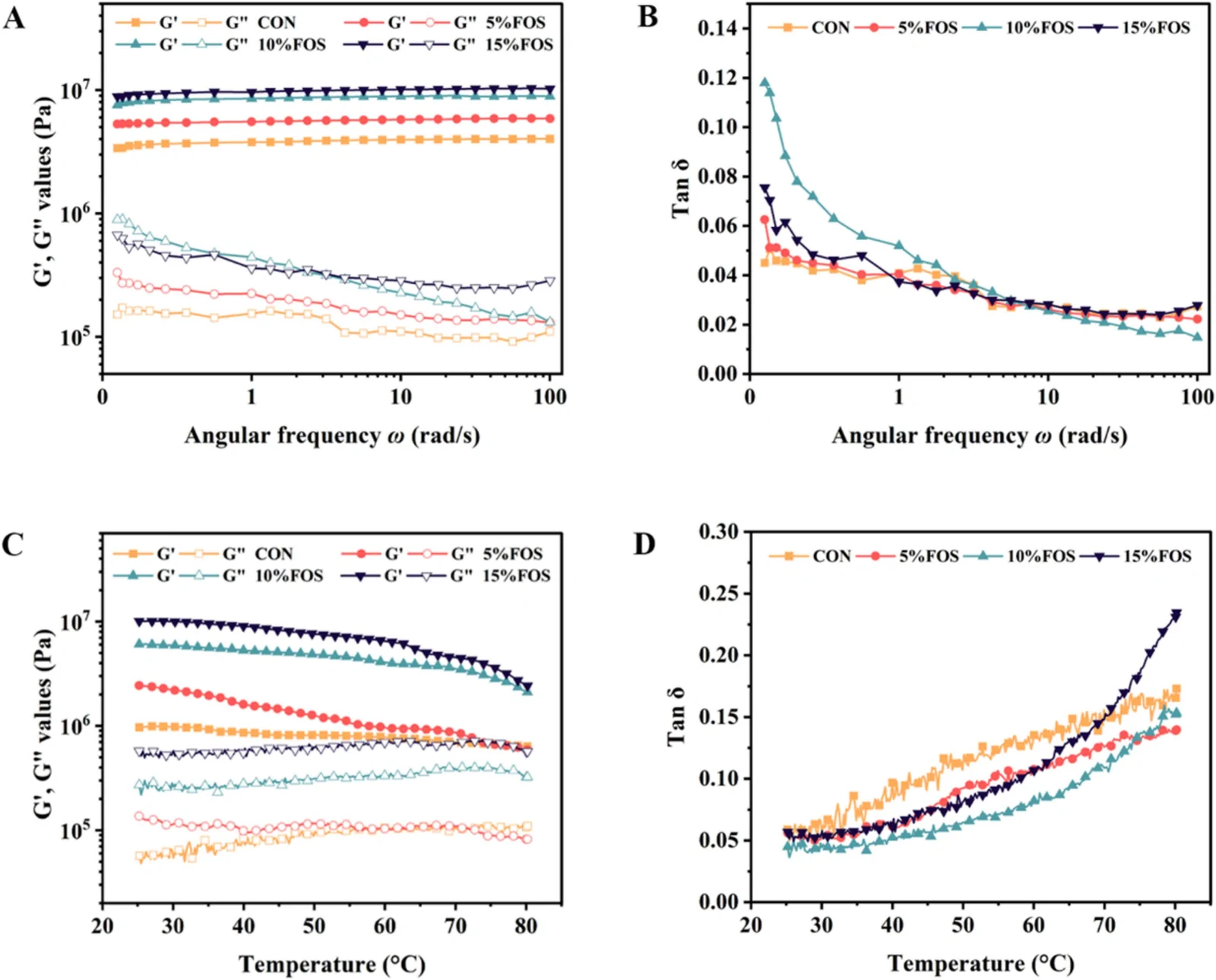
G', G“ and tan δ of frequency sweep (A, B) and temperature variation scanning (C, D) of the freeze-dried cheese supplemented with different levels of FOS. A and C: storage modulus (G') and loss modulus (G"); B and D: loss tangent (tan δ).

Frequency and temperature scans were used to evaluate the effect of FOS on the rheological properties of freeze-dried cheese. Frequency sweep results show that the storage modulus (G') of all samples increased with frequency and remained higher than the loss modulus (G"), indicating solid-like behavior (Zhang et al., 2025). The addition of FOS to the freeze-dried cheese greatly enhanced the storage modulus and loss modulus in the order of 15%FOS > 10%FOS > 5%FOS > CON, according to the dynamic viscoelasticity data in Fig. 6A. Higher G' and G" values may indicate a stronger structure, suggesting that the addition of FOS can lead to a more homogeneous texture and a denser structure, producing a stronger microstructural network. This enhancement is primarily attributed to the polyhydroxy structure of FOS, which enables it to form various interactions such as hydrogen bonds with proteins and water molecules. This strengthens intermolecular connections within the cheese, constructing a denser and more stable network structure. Previous studies have demonstrated that glycosylated ovalbumin exhibits stronger intermolecular forces, forming more robust bonded structures within emulsions (Lu et al., 2024). This enhanced intermolecular crosslinking is industrially beneficial, as it improves the product's resistance to fragmentation during handling and packaging.

Fig. 6C illustrates the dynamic viscoelastic changes of freeze-dried cheese samples with different proportions of FOS over the temperature range of 25 to 80 °C. Throughout the heating process, all samples consistently exhibited G' values higher than G". As previously reported by Zhang et al. (2025), plant-based cheese with added polysaccharides did not exhibit typical melting characteristics from solid to liquid even after exceeding 60 °C. Additionally, the G' and G" values of freeze-dried cheese increased with higher FOS concentrations. This suggests that a higher addition of FOS enhances the thermal stability of cheese, resulting in a more stable network structure with greater resistance to temperature changes. Sołowiej et al. (2023) investigated the effect of Pleurotus ostreatus polysaccharide addition on the rheological properties of casein-based processed cheese models. They reported that both G' and G" increased with rising Pleurotus ostreatus content in the samples. This improved thermal stability is particularly advantageous for commercial supply chains, as it helps maintain product quality during temperature-fluctuated storage and logistics.

Tan δ is the ratio of G" to G'. As shown in Fig. 6B and Fig. 6D, at low frequencies, the CON group exhibited the lowest tan δ, while the 10%FOS sample showed a notably higher value, indicating that FOS at this concentration acts mainly as a hydrated filler that increases viscous dissipation rather than forming a continuous elastic network. As frequency increased, the tan δ of the 10%FOS sample decreased and converged with the other groups, suggesting that under high-frequency deformation, the elastic response becomes more dominant and network differences become less apparent. Lower tan δ values reflect restricted molecular segment mobility and reduced molecular migration, conferring robust properties to the product (Ferrão et al., 2018). Notably, the 10%FOS group exhibited higher tan δ than the 15%FOS group at low frequencies. At lower concentrations, FOS molecules are dispersed as hydrated fillers within the casein network pores. Li et al. (2023) reported that adding 2–8% FOS increased system thixotropy, suggesting that at lower levels, FOS contributes mainly to intermolecular frictional dissipation—that is, viscous response. At 15%FOS, however, extensive hydrogen bonding between FOS molecules and the casein matrix creates an interconnected network. Silva et al. found that FOS enhances the elasticity and cohesiveness of biopolymer gels, with optimal gel structure and minimal porosity achieved at specific concentrations (Silva & Sato, 2017). This shift from a “hydrated filler“ to a “gel skeleton“ mode enhances G' disproportionately to G", lowering the G“/G' ratio and thus reducing tan δ. Simultaneously, none of the four cheese samples exhibited significant softening when heated to 80°C, with tan δ values remaining below 0.3 (G' > G”), though a gradual softening trend was observed with increasing temperature.

### 3.9 Color analysis

Table 3 presents the colorimetric analysis results of freeze-dried cheese supplemented with different amounts of FOS. The L* value, a critical parameter characterizing food whiteness, exhibited a significant positive correlation with FOS addition levels in this study. This result indicates that FOS can effectively enhance the brightness and whiteness of freeze-dried cheese. Previous studies have shown that the L* value is affected by light scattering from water molecules: tightly bound water molecules transmit less light, resulting in increased light reflection and higher whiteness. Conversely, a looser microstructure allows more light to penetrate, leading to lower whiteness (Cheng et al., 2024). The addition of FOS results in a more uniform and finer porous microstructure in freeze-dried cheese, thereby enhancing light scattering capacity and improving whiteness. The a* values remained negative across all samples, indicating that the addition of FOS did not alter the green tones of the cheese. In terms of b* values (yellow/blue axis), the results reflect that the addition of FOS increased the yellow color of the cheese. 15%FOS showed the most pronounced.

**Table 3:** Colorimetric analysis of the freeze-dried cheese supplemented with different levels of FOS.

Sample	Parameter		
	L*	a*	b*
CON	88.66 ± 0.44^c^	−0.20 ± 0.02^a^	10.86 ± 0.58^b^
5%FOS	90.61 ± 0.56^b^	−0.25 ± 0.03^b^	11.24 ± 0.18^b^
10%FOS	91.24 ± 0.12^b^	−0.30 ± 0.04^c^	11.58 ± 0.27^b^
15%FOS	93.01 ± 0.39^a^	−0.27 ± 0.02^bc^	12.35 ± 0.42^a^

1 Values are expressed as mean ± SD, *n* = 3.

2 a-d Values followed by different letters in the same column are significantly different (*p* < 0.05); values with the same letter are not significantly different.

### 3.10 Multivariate correlation network-based analysis of freeze-dried cheese quality correlations

#### 3.10.1 Relationship between FOS content and pore structure parameters of freeze-dried cheese

**Fig. 7:**
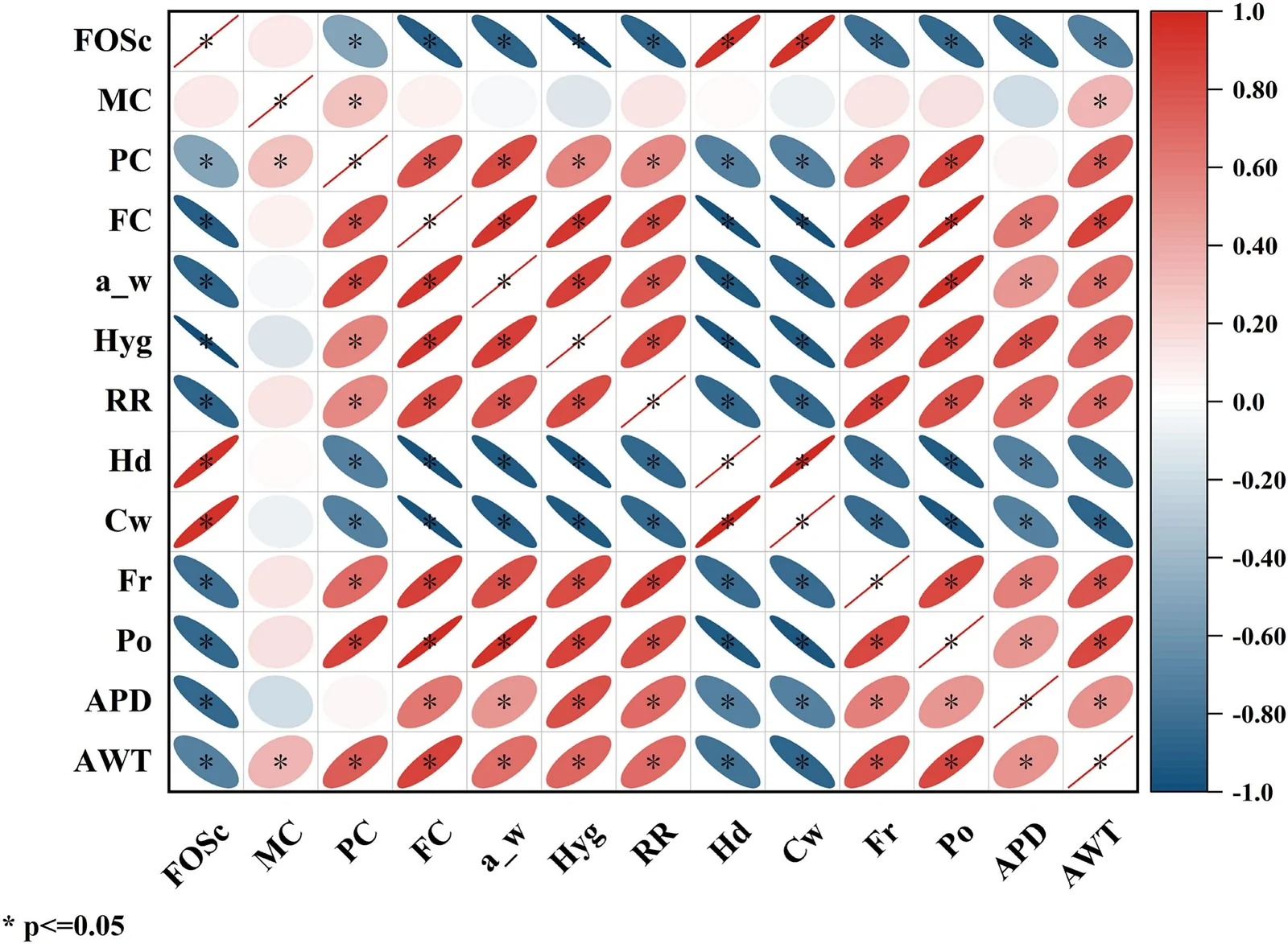
Correlation between intrinsic properties of freeze-dried cheese and corresponding freeze-drying characteristics. FOSc: Fructooligosaccharide content; MC: Moisture content; PC: Protein content; FC: Fat content; a_w: Water activity; Hyg: Hygroscopicity; RR: Rehydration ratio; Hd: Hardness; Cw: Chewiness; Fr: Fracturability; Po: Porosity; APD: Average Pore Diameter; AWT: Average Wall Thickness.

FOS content shows negative correlations with porosity, average pore diameter, and average wall thickness (*r* = −0.85, −0.86, −0.71, respectively), whereas smaller pore diameters were associated with thinner walls (*r* = 0.52) (Fig. 7). This strong association indicates that FOS content is linked to pore structure refinement, which is characterized by smaller pores, thinner walls, and a denser network. Earlier reports have shown that small-molecule sugars in freeze-drying systems can inhibit ice crystal size by altering the water state, yielding finer pore structures after sublimation (Feng, Bi, Yi, Li, Li, & Ma, 2022).

The observed pore-structure features are plausibly linked to the dual effects of FOS on ice crystal nucleation and growth during freezing. The hydrophilic hydroxyl groups of FOS can surround and immobilize water molecules via hydrogen bonds, acting as active sites for heterogeneous nucleation (Sun et al., 2022). This provides an ordered template for water molecules, promoting the formation of fine, homogeneous pores. Guerreiro et al. (2020) proposed that polysaccharides enhance polymer chain hydration and participate in water-ice interfacial interactions, which may explain this role. During ice crystal growth, FOS reduces the growth rate by altering diffusion properties (Feng, Bi, Yi, Li, Lyu, et al., 2022): compared with pure water, water molecules bound to FOS hydroxyl groups have a lower self-diffusion coefficient (Chen et al., 2018). FOS forms a strong hydrogen-bonding network with water via equatorial hydroxyl groups, creating “hydrated saccharide clusters” that hinder bulk water diffusion and reduce molecular mobility. This kinetic constraint, combined with abundant binding sites on ice crystal surfaces, increases the volume of the unfrozen liquid microphase and enhances liquid-phase bridges between crystals. Ultimately, the system produces fine, uniformly distributed ice crystals, whose structural strength manifests as a thin-walled, micropore-dominated porous skeleton (Gonda & Sei, 2005).

#### 3.10.2 Relationship between FOS content and textural properties of freeze-dried cheese

As shown in Fig. 7, FOS content was positively correlated with hardness and chewiness (*r* = 0.96 and 0.95, respectively). This positive correlation suggests that increased FOS content is associated with greater hardness and chewiness. Previous work has confirmed a significant negative correlation between pore size and compressive strength in porous structures (Zhao et al., 2018), consistent with the negative correlation we observed between average pore size and hardness (*r* = −0.71). The correlation coefficient between FOS content and fracturability was −0.81, indicating an inverse relationship between FOS addition and fracturability. Products with larger and more abundant pores tend to be crispier, whereas those with fewer and smaller pores are harder and less brittle (Feng et al., 2024). This explains the positive correlation between average pore diameter and fracturability (*r* = 0.58). These texture-related correlations further highlight the key role of FOS in modulating the physical properties of freeze-dried cheese.

#### 3.10.3 Relationship between FOS content and the hygroscopicity and rehydration ratio in freeze-dried cheese

Fig. 7 indicates that FOS content is negatively correlated with hygroscopicity and rehydration ratio (*r* = −0.99 and − 0.87, respectively). Thus, FOS addition significantly reduced both parameters. Pore structure is the key pathway mediating this reduction. Correlation analysis revealed a very strong negative correlation between FOS content and hygroscopicity (*r* = −0.99, *p* < 0.05), which aligns closely with the strong negative correlations between FOS content and both porosity and average pore diameter (*r* = −0.85 and − 0.86, *p* < 0.05). More importantly, both porosity and average pore diameter showed significant positive correlations with hygroscopicity (*r* = 0.87 and 0.81, *p* < 0.05). This suggests that the refined pore structure associated with FOS addition may inhibit capillary action and hinder the infiltration of external moisture (Hu et al., 2024; Nimmo, 2004), thereby correlating with lower hygroscopicity.

The denser pore network also appears to account for the observed decrease in rehydration capacity. Rehydration behavior primarily depends on the ability of the dried matrix to capture and retain water. FOS content showed a significant negative correlation with the rehydration ratio (*r* = −0.87, *p* < 0.05), while the rehydration ratio was highly positively correlated with both porosity and average pore diameter (*r* = 0.80 and 0.69). This quantitative relationship supports the interpretation that the denser, smaller-pore structure associated with FOS addition makes it more difficult for water molecules to rapidly enter and fill the interior of the solid matrix, ultimately leading to a lower rehydration ratio (Zhang et al., 2024). It is worth noting that these correlation analyses were based on only four FOS addition levels, so the limited number of data points may affect the robustness of the observed relationships. Nevertheless, the consistent trends across multiple parameters support the proposed mechanistic interpretation.

This study offers practical guidance for formulating FOS-fortified fresh cheese. Addition of 5–15% FOS effectively improves texture and water distribution, with the 5–10% range providing a favorable balance between quality improvement and cost control—a dosage that also falls within current food additive regulations. Several limitations should be acknowledged, however. Quality assessment was based on instrumental measurements only, without sensory verification; storage performance was evaluated over a short term only; and the fate of FOS during processing was not quantitatively tracked. Moreover, given the limited number of experimental batches and the absence of pilot-scale trials, the optimal 10%FOS level identified here is strictly applicable to unfermented fresh cheese. Future work will focus on optimizing processing parameters and ingredient mixing uniformity to minimize FOS degradation, enhance consumer acceptability, and support scalable production.

## 4 Conclusions

Overall, FOS addition markedly improves the quality of freeze-dried cheese, manifested as a refined microstructure, enhanced texture, and increased whiteness. These improvements are consistent with the hypothesis that hydrogen-bonding interactions among FOS, casein, and water modulate water mobility and ice crystal growth during lyophilization, yielding a denser and more uniform porous network with better thermal stability and mechanical strength. However, this benefit is dose-dependent: while 15%FOS provided the highest mechanical performance, it also led to a dilution of protein and fat content on a dry matter basis, a marked reduction in rehydration ratio. Therefore, considering both the quality enhancements and the potential limitations, 10%FOS was identified as the optimal level. This formulation not only reduced water activity to a favorable range and lowered hygroscopicity, but also delivered a firm texture and an acceptable balance of composition and rehydration properties, while also providing a more cost-effective formulation compared to the 15%FOS group. The proposed mechanistic pathway is plausible and supported by our data, yet the exact molecular regulation warrants further investigation. Collectively, judicious FOS supplementation offers a practical strategy for producing premium freeze-dried cheese.

## CRediT authorship contribution statement

**Yang Lyu:** Writing – review & editing, Writing – original draft, Supervision, Resources, Methodology, Funding acquisition, Formal analysis. **Shengnan Li:** Resources, Methodology, Investigation, Conceptualization. **Lei Gao:** Resources, Methodology, Data curation. **Zijian Zhao:** Resources, Methodology, Data curation. **Hao Qian:** Resources, Formal analysis. **You Kang:** Resources, Formal analysis. **Fei Chen:** Investigation, Data curation. **Xindong Jia:** Investigation, Data curation.

## Declaration of competing interest

The authors declare that they have no known competing financial interests or personal relationships that could have appeared to influence the work reported in this paper.

## Data Availability

Data will be made available on request.
